# A Review of Phosphate Mineral Nucleation in Biology and Geobiology

**DOI:** 10.1007/s00223-013-9784-9

**Published:** 2013-09-28

**Authors:** Sidney Omelon, Marianne Ariganello, Ermanno Bonucci, Marc Grynpas, Antonio Nanci

**Affiliations:** 1 Chemical and Biological Engineering, University of Ottawa, Ottawa, Canada; 2 Faculty of Dentistry, Université de Montréal, Montreal, Canada; 3 Department of Experimental Medicine, La Sapienza University of Rome, Rome, Italy; 4 Laboratory Medicine and Pathobiology, Samuel Lunenfeld Research Institute of Mt. Sinai Hospital, Toronto, Canada

**Keywords:** Biomineralization: mechanisms, Bone and cartilage development, Crystal structure/crystallinity

## Abstract

Relationships between geological phosphorite deposition and biological apatite nucleation have often been overlooked. However, similarities in biological apatite and phosphorite mineralogy suggest that their chemical formation mechanisms may be similar. This review serves to draw parallels between two newly described phosphorite mineralization processes, and proposes a similar novel mechanism for biologically controlled apatite mineral nucleation. This mechanism integrates polyphosphate biochemistry with crystal nucleation theory. Recently, the roles of polyphosphates in the nucleation of marine phosphorites were discovered. Marine bacteria and diatoms have been shown to store and concentrate inorganic phosphate (Pi) as amorphous, polyphosphate granules. Subsequent release of these P reserves into the local marine environment as Pi results in biologically induced phosphorite nucleation. Pi storage and release through an intracellular polyphosphate intermediate may also occur in mineralizing oral bacteria. Polyphosphates may be associated with biologically controlled apatite nucleation within vertebrates and invertebrates. Historically, biological apatite nucleation has been attributed to either a biochemical increase in local Pi concentration or matrix-mediated apatite nucleation control. This review proposes a mechanism that integrates both theories. Intracellular and extracellular amorphous granules, rich in both calcium and phosphorus, have been observed in apatite-biomineralizing vertebrates, protists, and atremate brachiopods. These granules may represent stores of calcium-polyphosphate. Not unlike phosphorite nucleation by bacteria and diatoms, polyphosphate depolymerization to Pi would be controlled by phosphatase activity. Enzymatic polyphosphate depolymerization would increase apatite saturation to the level required for mineral nucleation, while matrix proteins would simultaneously control the progression of new biological apatite formation.

## Introduction

In nature, different calcium phosphate minerals are produced within a wide range of environments by geological (igneous apatite), geochemical and/or geomicrobiological (phosphorite), and biological (biological apatite) processes. Igneous apatite minerals nucleate and crystallize from molten, phosphate-rich rock, forming crystalline fluorapatite (Ca_5_F_2_[PO_4_]_3_), chlorapatite (Ca_5_Cl_2_[PO_4_]_3_), or hydroxyapatite (Ca_5_[OH]_2_[PO_4_]_3_) [1]. In less extreme environmental conditions, biochemical pathways are attributed to the precipitation of biological apatite and phosphorite (minerals that contain >6 % P on a dry basis [2]) within aqueous environments at neutral to basic pH.

Phosphate concentrations in environmental (e.g., marine), intracellular, or extracellular (e.g., osteoid) aqueous and calcium-containing environments are too low for spontaneous, inorganic formation of a first calcium phosphate mineral from solution (nucleation). However, calcium phosphate minerals do nucleate and grow in these environments. The chemical mechanism of phosphorite mineral nucleation in the environment has been a question for over 100 years, as has the chemical mechanism of biological apatite nucleation within organisms.

The process of apatite biomineralization was proposed to be conserved over many eras and over a wide range of life forms. Thompson [3] quoted J. H. Mummery, who in 1914 wrote “Calcification in both dentine and enamel is in great part a physical phenomenon; the actual deposit in both occurs in the form of calcospherites, and the process in mammalian tissue is identical in every point with the same processes occurring in lower organisms.” This potentially evolutionarily conserved mechanism has allowed for the formation of elegant structural and metabolically active apatitic skeletons for a wide range of vertebrates and some invertebrates that was poetically described by Quekett:

The laws of Nature are undeviating in the construction of the skeleton of vertebrate animals: the same regularity in structure, the same method of arrangement of the bone-cells, has existed from the time when the surface of our planet was first inhabited by a vertebrate animal up to the present period. The largest bones of the mighty Iguanodon (say of 100 feet in length), of the Ichthyosaurus—the tyrant of the water in former ages, of the gigantic Tortoise of the Himalaya range (some 20 feet in length), present no appreciable difference, in their minute structure, from the pigmy race of lizards that we now tread under our feet. The bones of the Mastodon and the huge Megatherium, the giants of the land, are no more remarkable for the coarseness of their structure than are those of the smallest of the mammiferous quadrupeds, the mouse, and such has been the prevailing law from the commencement of the earth’s existence, and such, no doubt, will continue to the end of time [4].

This review summarizes recent advances in understanding the chemical mechanisms of phosphorite biomineralization induced by organisms much smaller than mice. Sulfide-oxidizing marine bacteria and diatoms have been discovered to share a common mechanism of inorganic phosphate (Pi) concentration and storage. The geobiology community has reported that low environmental Pi concentrations can be concentrated and stored by biochemical polymerization of Pi into polyphosphate ([PO_3_^−^]_n_, polyP) molecules [5]. Pi release from polyP can occur through controlled phosphatase hydrolysis, or through uncontrolled hydrolysis in the environment. Either of these pathways results in an increase in local Pi concentration, which increases phosphate mineral saturation. This increase in phosphate mineral saturation may allow for spontaneous phosphate mineral nucleation in the environment, or within organisms.

This article will provide a brief summary of polyP chemistry, phosphorite and apatite mineralogy, as well as the chemistry of mineral nucleation. This will provide a background for reviewing two phosphorite mineral nucleation pathways, and a commentary on the similarities of phosphate mineral nucleation reported in the fields of geobiology, dentistry, and biology.

## PolyP is Both a Mineralization Inhibitor and a Bioavailable Pi Source

In the skeletal mineralization research community, polyPs are noted to be “highly inhibitory to calcium phosphate nucleation and precipitation” [6]. Francis [7] demonstrated that polyPs inhibit crystalline calcium hydroxyapatite nucleation and growth from solution as long as they are intact. Temperature, pH, and some enzymes enhanced their hydrolytic instability, decreasing the polyP concentration and therefore reducing their inhibitory activity.

Fleisch et al. [8] studied the mineralization inhibitor effect of pyrophosphates (P_2_O_7_^4−^) and polyPs on the mineralization of chick embryo femurs grown in culture. Normalized to 4 and 16 μg phosphorus (P)/mL, polyP inhibited mineralization. However, 1 μg P/mL “seemed to activate calcification,” which was not observed in their previous in vitro studies [6]. They attributed this to the possibility that phosphatase enzymes may have cleaved the phosphate esters, destroyed the inhibitory molecules, and that this “might lead to facilitation of calcium phosphate deposition” [8]. Fleisch and Neuman [6] noted that “ossifiable cartilage and bone contain enzymes which destroy polyphosphates” and suggested that the colocation of polyPs and phosphatase enzymes may remove mineral inhibition and “activate calcification.” What they did not discuss, and has recently been accepted in the geobiology literature, is that destruction of polyP produces Pi. While complete polyP destruction removes the inhibitor, at the same time it increases Pi concentration. In the presence of free calcium ions, this increases the chemical potential for nucleating calcium phosphate minerals. Much as glucose concentration can be controlled by glycogen formation and destruction, Pi concentration can be controlled by polyP formation and destruction. Interest in inorganic polyP biochemistry is growing [9] and has provided interesting possibilities for enzymatic control of free Pi concentration.

PolyPs (metaphosphates) were identified within yeast in 1936 [10]. In the 1940s, yeast was observed to concentrate P as polyP within volutin granules after periods of Pi starvation [11–13]. The presence of polyPs was further confirmed within yeast cells in 1975 by ^31^P NMR [14]. In 1980, the fluorescent dye 4′,6-diamidino-2-phenylindole (DAPI), typically used to stain DNA, was used to identify intracellular polyP within volutin granules [15]. The amplified blue emission of the excited DAPI–DNA complex has a maximum intensity at ~340 nm, while the DAPI–polyP complex emission is yellow-green (maximum intensity at ~526 nm) [15]. PolyP identification methods have different deficiencies [16]. For example, histological stains of polyP are not specific, the DAPI–RNA complex fluoresces at 500 nm [17], and samples often require processing to concentrate polyP for detection with ^31^P NMR. These sample preparation methods can remove or break down polyP.

PolyPs are negatively charged polyanions with great affinity for calcium and other multivalent cations [18]. PolyP chelation of calcium reduces the free calcium concentration [19] and produces a neutral, amorphous, [Ca(PO_3_)_2_]_n_ complex. This complex is a bioavailable reserve of Ca^2+^ and Pi. The formation of these complexes has been attributed to mitochondria; their production and metabolism in mammalian cells have been investigated [20]. The effect of polyP on yeast and animal cell mitochondrial functions and dysfunctions [21] and polyP roles in biochemistry [9] have been recently reviewed [22].

## PolyP is a Bioavailable Pi and Calcium Storage Strategy

Using electron microscopy, electron-dense granules were observed in rat liver mitochondria from a calcium and Pi accumulation study in 1964 [23]. These mitochondrial, electron-dense granules contained high calcium and Pi concentrations (reported to be “at least 0.5 M” when Pi was measured by the Fiske-Subbarow method [24] and 0.8 M by Lehninger in 1970 [25]). However, these granules were surprisingly amorphous [23]. At these high Pi concentrations, a calcium phosphate mineral was expected to nucleate. However, if the P was assumed to be from polyP, which offers a higher P density than Pi [26], then the structure of the calcium- and P-rich granule would be expected to be amorphous. Amorphous calcium–polyP requires very high temperatures to crystallize [27].

Discrete granules containing calcium–polyP complexes have been proposed to be conserved from bacteria to humans [28]. Theoretically, the Ca:P ratio of this complex is less than 1; the exact value is a function of the polyP chain length. The neutral Ca–polyP complex represents a concentrated, bioavailable calcium and P store as polyP destruction produces Pi and frees Ca^2+^. At neutral to basic pH, this polyP depolymerization simultaneously removes an apatite mineralization inhibitor and increases apatite saturation—the chemical potential for apatite nucleation from solution.

Although polyP depolymerization is thermodynamically favored in aqueous environments, the kinetics are slow at neutral pH [29], but accelerated by phosphatase enzymes such as alkaline phosphatase (APase) [30]. PolyP is a substrate for both tissue-nonspecific APase [31] and intestinal APase [32]. APases cleave Pi from ester phosphates at neutral to basic pH [33] and are theorized to cleave Pi from polyP in diatoms [34].

The relationship between Ca and P concentration and storage as Ca–polyP granules, and the geobiological production of phosphorite mineral from these concentrated stores in bacteria and diatoms will be described. This will be compared with a review of Ca- and P-containing granules and APases identified in protists, brachiopods, and vertebrates that control biological apatite mineralization.

## Apatite, Phosphorite, and Biological Apatite Minerals

The chemical and physical characteristics of minerals lay the foundation for explaining the processes that form them. The family of apatite minerals is defined with the generalized formula A_5_(XO_4_)_3_Z, where A is a divalent cation that is most often Ca, X is most commonly P, and Z represents an anion, which can be one or more of F, Cl, and OH [1]. Apatite minerals are very tolerant of elemental and molecular (e.g., HPO_4_^2−^, CO_3_^2−^) substitutions, so the apatite group is large, with over 25 members [35]. This chemical diversity means that exact analysis of apatite mineral samples is challenging.

Geological phosphorite is “composed essentially of carbonate apatites which are usually moderately high in fluorine” [36]. Although their chemical definition is similar to bone mineral, phosphorites vary in chemical composition, containing apatite and carbonated fluorapatite [37–39] and vary in mineralogy [40]. Phosphorite rock generally describes a group of sedimentary (deposited by water, ice, or wind) rock deposits with high P concentration [41]. Phosphorites have been described as “sedimentary deposits with high phosphorus concentration” [18] and minerals with a lower limit of >9 % PO_4_^3−^ [42] because these deposits contain phosphate rocks of different mineralogies [40]. Many phosphorite formation theories have been proposed, including inorganic precipitation and biomineralization processes [43].

Biological apatite minerals are formed within vertebrate skeletal tissues, within inarticulate brachiopods (order Atremata, superfamily Lingulacea, and order Neotremata) [44–46] and the protozoa *Spirostomum ambiguum* [47]. The vertebrate skeletal mineral was identified as containing calcium, phosphate, and carbonate in 1894 [48] and further described as a poorly crystalline carbonated apatite mineral in 1927 [49]. In 1929, the fluoride component of bone mineral was identified [50]. Since then, mineralogists have described bone mineral as a substituted carbonated apatite similar to dahllite (an apatite mineral with a fluoride content <1 %) [51, 52]. A proposed structural formula for bone mineral is (Ca,X)_10_(PO_4_,CO_3_,Y)_6_(OH,Z)_2_ with X substituting cations and Y, Z substituting anions (with the stoichiometry changing accordingly) ([53] citing [54–57]). Bone apatite is consistently nanometer-scaled [58]; is less crystalline than highly crystalline, synthetic hydroxyapatite [59]; contains carbonate and fluoride [50]; is highly substituted [36]; and contains a small fraction of the hydroxyl groups expected in pure hydroxyapatite [60, 61]. Since Neuman and Neuman [62] stated that “the hydroxy apatite is the only solid phase of the Ca-PO_4_-H_2_O system which is stable at neutral pH,” the literature has mistakenly described the mineral in bone as “hydroxyapatite”. Unlike phosphorite, the consistent size and chemistry of skeletal mineral suggest that its nucleation is a highly controlled process.

Bone mineral growth is predicted from the calcium and phosphate concentrations in serum, but these concentrations do not explain bone mineral nucleation. How biological apatite first nucleates within the extracellular matrix (ECM) is still an open question, and must involve the active participation of matrix proteins. As described by McConnell [36], “increments in the organic and inorganic chemistries can be isolated for separate consideration, and factors which interrelate these systems are being sought.” Ultimately controlled by biochemical processes, biological apatite mineral nucleation should also follow the physical chemistry principles which describe how minerals nucleate from solution.

## Mineral Saturation States and Nucleation

A mineral nucleates from solution if the mineral saturation state is above the equilibrium (saturated) value. The equilibrium saturation value at a given temperature is also termed the “solubility product” and is reported as *K*_sp_ for pure minerals. The mineral saturation state is proportional to the activities of its component ions, raised to the power of their stoichiometric coefficients (ion activity product [IAP] [63]). If the IAP is larger than the mineral *K*_sp_, the solution is “supersaturated” with respect to that mineral. The degree of supersaturation dictates the possibility of crystal growth (lowest supersaturation), heterogeneous nucleation (intermediate supersaturation), or homogeneous nucleation (highest supersaturation) [64]. The IAP of different calcium phosphate minerals at different pH values was reported by Larsen [65].

Fleisch and Neuman [6] noted that “extracellular fluids are supersaturated with respect to bone mineral, and that the concentrations of calcium, Pi, and hydroxyl ions are sufficiently high to support the growth of bone mineral crystals once the initial crystals have formed.” After describing this low supersaturation state that explains observed bone crystal growth, they continue, “however, the concentrations of these ions are not high enough to precipitate spontaneously. Some seeding mechanism seems required to initiate crystallization ” (referencing [62]). Spontaneous precipitation is termed “homogeneous nucleation” and requires the largest supersaturation values. Nucleation on another solid phase requires a lower supersaturation value. Collagen was proposed to be the solid phase for bone mineral nucleation, resulting in a lower apatite supersaturation value required for heterogeneous nucleation of the ECM [66].

This theory of heterogeneous nucleation of apatite on collagen was termed the “eptitactic” theory [67]. However, the physiological concentrations of calcium and Pi [68] are not high enough to induce heterogeneous precipitation on collagen [6] or from solution [69]. In 1923, Robison [70] suggested that an enzymatically controlled Pi-concentration increase would be possible by cleaving “an organic ester of phosphoric acid.” One candidate enzyme for this activity in bone tissue is APase, which is associated with apatite biomineralization [71]. Geochemists and geomicrobiologists have similarly attributed inorganic and biological phosphorite precipitation to a discrete, local increase in Pi concentration.

## Phosphorite Nucleation

Kazakov [72] reviewed the theories for geological phosphorite formation processes in 1937. He surveyed the range of theorized mechanisms, including inorganic precipitation, biological (“biolitic”) formation by plankton, residual skeletons (“necton”) that settle on the ocean floor, and organisms that live at the bottom of the ocean (“bentos”), referencing papers from the late 1800s and early 1900s. There are some examples of inorganic processes, such as mixing waters of different compositions in the environment, which will increase phosphorite saturation. The simplest inorganic phosphorite precipitation process is mixing calcium-rich waters (e.g., seawater or limestone deposit pore waters) with phosphate-rich waters (e.g., deep sea upwelling or rainwater that has percolated through guano deposits). If the resulting mixed solution is supersaturated with respect to phosphate mineral at neutral or basic pH, then the chemical potential exists for homogenous and/or heterogenous calcium phosphate mineral nucleation.

Another P source from geological aqueous environments comes from sudden and local P fluxes from sediments which are coincident with anaerobic conditions. In 1912, it was proposed that under “conditions probably anaerobic but not yet well understood . . . phosphoric acid also liberated will react with various substances, particularly lime salts. With the latter, it produces the mineral collophanite, a hydrous calcium carbo-phosphate” ([73] citing [74]). Since the 1930s, this inorganic process of Pi release was related to the reduction of Fe(III) to Fe(II). This chemical reduction of iron dissolves Fe(II) and releases the Pi that was adsorbed to Fe(III) hydroxide solids that dissolve in the anoxic sediment ([75] citing [76], [77]). It was later proven that organisms can concentrate and store Pi from their environment [78] and then release Pi to survive periodic anaerobic environments [79] or after death [75, 80]. This biologically driven Pi release can increase phosphorite saturation in the vicinity of the Pi-releasing organisms. This is an example of biologically induced mineralization [81] as the mineralization occurs in the environment around the organism as an indirect consequence of biological activity. Because the conditions of this environment may change, the biologically induced phosphate minerals may not be consistent in size, composition, or mineralogy, which is characteristic of phosphorite minerals.

## Biologically Induced Apatite Nucleation: Benthic Bacteria

The “biologic” theory, proposing that life-forms play a role in phosphorite formation, was published in 1936 [82]; but the exact biochemical mechanisms were unknown at that time. More recently, Pi-accumulating marine bacteria *Pseudomonas* and *Acinetobacter* were thought to be involved in modern (meaning still actively forming) phosphorite formations [83]. This was proposed because similar bacterial species accumulate Pi as polyP in biological activated sludge systems used to treat wastewater ([83] citing [84]). These bacteria concentrate Pi as polyP in oxic conditions; when their environment becomes anoxic, they depolymerize polyP as an energy source, releasing Pi. This switch in energy production was proposed to explain the local Pi concentration increase in the calcium-rich marine pore water, leading to apatite precipitation. Marine bacteria above modern phosphorite deposits were collected, and intracellular polyPs were identified with chemical fractionation methods [83].

Modern phosphorite formation has also been associated with two species of sulfide-oxidizing bacteria that inhabit microbial mats within the marine sediment: *Beggiatoa* [85, 86] and *Thiomargarita namibiensis* [79, 87]. In situ Pi concentrations of up to 300 μM were measured within ocean sediment composed of ~25 % hydroxyapatite and populated by *Thiomargarita* [79]. This represents a remarkable increase in Pi concentration as open-ocean Pi concentrations are generally less than 1 μM [88]. Incubation of these bacteria in the laboratory demonstrated Pi release under anoxic conditions [79]. It was concluded that polyP hydrolysis caused the increase in marine sediment pore water Pi concentration, leading to biologically induced hydroxyapatite precipitation [79].

**Fig. 1:**
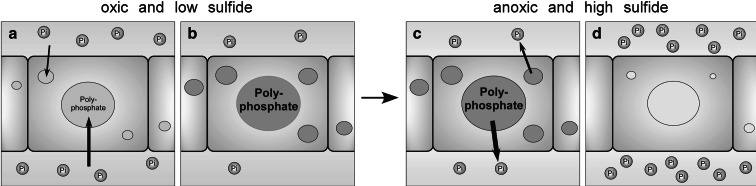
Proposed phosphate uptake and release by *Beggiatoa*. **a**, **b** Under oxic conditions and exposure to low sulfide concentrations, phosphate is taken up by *Beggiatoa* and accumulated as polyphosphate. The phosphate concentration in the medium decreases. **c**, **d** When the conditions change to anoxia and exposure to sulfide increases, the *Beggiatoa* decompose polyphosphate and release phosphate. This leads to an increase in phosphate in the medium. Reprinted by permission from Macmillan Publishers Ltd: ISME J [89], copyright 2011

Goldhammer et al. [86] used ^33^P to trace the path of Pi from an in vitro aqueous medium into organic-rich sediments obtained from a modern phosphorite location that included *Thiomargaria* and *Beggiatoa*. They noted that under anoxic and oxic conditions these sulfide-oxidizing bacteria accumulated ^33^P, with more accumulation in oxic conditions and no detectable ^33^P incorporation into the dead (control) cells. Authigenic (locally formed) apatite containing ^33^P formed most rapidly under anoxic conditions, while no apatite was formed in the control experiments with dead cells. The authors concluded that authigenic apatite formation required the presence of living cells such as *Thiomargarita* and *Beggiatoa*, and that these cells accumulated phosphate as polyP and released Pi under anoxic conditions [86]. Brock and Schulz-Vogt [89] cultured *Beggiatoa* and concluded that Pi release from intracellular polyP stores was associated with anoxia and increasing sulfide concentrations (Fig. 1).

This mechanism of biological concentration of Pi as polyP and release to nucleate phosphorite gave rise to the recent comment in a microbiology review that “It seems that we are in the midst of a revolution in our understanding of the origins of phosphatic mineral deposits” [5]. The review describes the “compelling mechanism” for concentrating P as polyP within marine sulfide-oxidizing bacteria that live at the bottom of the ocean, and how polyP is a source of Pi that is released into the marine sediment pore waters. Also noted were the many gaps that remain in the detailed understanding of how this biological Pi-release process results in apatite precipitation.

The role of bacteria in apatite mineralization may not be limited to geological environments. A 1967 editorial titled “Microbiologic Calcification: Bone Mineral and Bacteria” by Ennever and Creamer [90] interpreted and expanded on the work by Bulleid in 1925 [91]. Their conclusion was that he had observed apatite mineralization caused by the action of the dental bacteria *Bacterionema matruchotii*.

## Biologically Induced Apatite Nucleation: Oral Bacteria

There are many types of oral bacteria within dental plaques; some of them are correlated with dental calculus formation. Dental calculus includes a range of calcium phosphate minerals, including apatite, Whitlockite, tricalcium phosphate, and octacalcium phosphate [92–95]. To test if the aqueous oral environment provided the chemical driving force (IAP) for calculus formation, Poff et al. [96] surveyed patients with a wide range of supragingival calculus severity and compared the calculus severity to the measured hydroxyapatite saturation in their saliva. The results showed no correlation, suggesting that inorganic calcium phosphate nucleation was not the mechanism of calculus formation. Poff et al. also discussed the possible role of mineral nucleation and growth inhibitors such as pyrophosphate [97] and polyphosphate [7], but concluded that they did not affect the study conclusions.

Bacteria are known to contribute to dental calculus, although their specific role is not clear. Microbes encased in mineral result in a porous calculus structure [98], suggest extracellular mineralization occurs. The varied mineral composition in dental calculus may reflect the varied environment in which induced biomineralization takes place [81]. The mechanism for these calcification events has not yet been explained.

Dental calculus formation and intracellular mineralization have been associated with the oral microbe *Bacterionema matruchotii*. Ennever et al. [99] reviewed their work on this organism [90], explaining that apatite was identified in the cell-ash in 20-day-old cells and identified intracellular apatite mineral within fixed and embedded cells [100]. A survey of oral bacteria concluded that many, including *Bacterionema matruchotii*, contained “dark-staining, electron-dense granules. . . 15 to 500 nm in diameter, probably polyphosphate granules” [101]. Takazoe and Nakamura [102] identified “metachromatic granules and intracellular calcification of *Bacterionema matruchotii*” and reported that the granules were “chiefly polymetaphosphate.” They used both measurement of the metachromasy of toluidine blue and chemical analysis of the extracted granules to confirm their polymetaphosphate conclusion. They observed the absorption maximum of toluidine blue shift when exposed to the extract, and the destruction of the metachromatic extract by an acidic treatment. They also measured an increased Pi concentration in the extract after acid hydrolysis. They noted that polyP is a mineralization inhibitor, and that it might have an inhibitory effect against dental calculus formation [102]. Dentifrices containing polyP have since proven to reduce oral bacteria adherence [103, 104] and to decrease dental caries formation [105].

The association of exogenous polyPs with mineral inhibition may have prevented Takazoe and Nakamura [102] from associating these intracellular granules within *Bacterionema matruchotii* as a Pi source for phosphate mineral formation. The identification of polyP within *Bacterionema matruchotii*, and the association of APase activity with bacteria of dental plaque [106, 107] provide two clues to a possible chemical mechanism for increasing calcium phosphate mineral supersaturation within oral microbial biofilms.

Could a parallel be drawn between induced phosphorite nucleation by bacterial Pi release into marine bacterial mats from intracellular polyP, and calculus nucleation by oral bacterial Pi release into biofilms from intracellular polyP stores? This mechanism could explain how calcium phosphate mineral supersaturation is increased within the oral biofilm, causing calcium phosphate mineral nucleation. This induced biomineralization process within the biofilm environment would also explain the varied dental caries mineralogy. Direct evidence of this proposed induced apatite biomineralization process by oral bacteria was not identified in the literature. The recent literature does provide a second biologically induced, phosphorite-formation mechanism in the deep ocean that involves polyP.

## Apatite Nucleation from PolyP Granules: Diatoms

In 1936, Cayeux [108] proposed that algae were responsible for the phosphorite deposits colocated with siliceous rock deposits. In the 1960s, algae were also noted for their ability to accumulate Pi from their environment. In the laboratory, *Phaeodactylum tricornutum* reduced Pi concentrations from its surroundings to less than 7.2 × 10^−10^ M [109]. In chlorococcalean algae, granules containing polyP stained with toluidine blue [110]. This staining method was similarly used to conclude the presence of polyP granules within the diatom *Melosira varians* [111]. The authors theorized that the polyP granules were a bioavailable Pi store, as it is one of the many proposed biological roles of polyP within unicellular organisms [112]. The validity of the toluidine blue staining method for polyP in algae was validated in 1996, when electron-dense bodies containing calcium and polyP were identified in *Chlamydomonas eugametos* with ^31^P NMR and DAPI. This study also colocated Ca and P, with Ca:P ratios <1, by X-ray microanalysis [113].

Phytoplankton are theorized to produce and secrete APase into their environment in order to release Pi from dissolved organic phosphorus, and increase the local Pi concentration [114]. In 2012, Dyhrman et al. [34] identified cell surface–associated APase, suggesting that Pi hydrolysis and Pi uptake may be “tightly coupled.” They also identified polyP polymerase regulation, with cellular P condensing to polyP, and related their results to the relationship between diatoms and marine phosphorite mineral formation identified by Diaz et al. in 2008 [80].

**Fig. 2:**
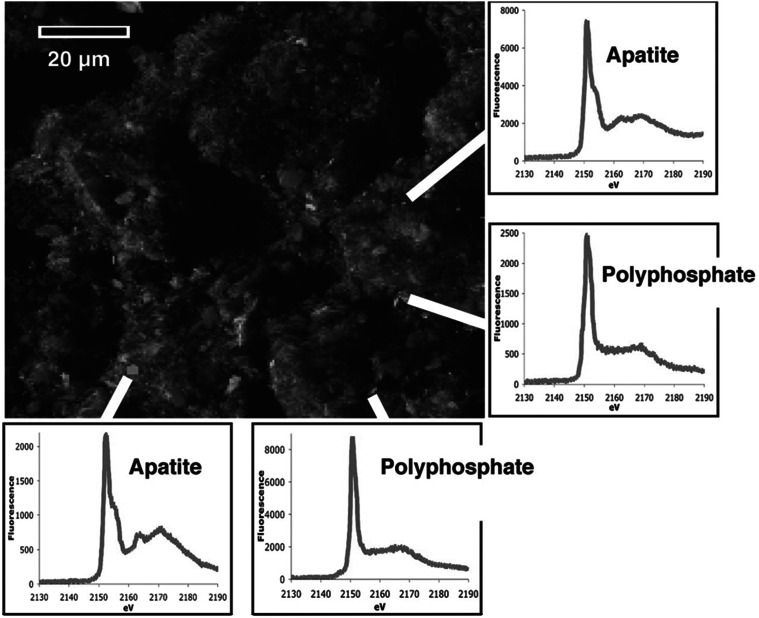
X-ray fluorescence micrograph and fluorescence spectra of phosphorus-rich regions in Effingham inlet sediment. Sedimentary phosphorus (*red*) appears as distinct, heterogeneously distributed submicrometer-sized particles against a comparatively uniform background of sedimentary aluminum (*blue*) and magnesium (*green*). On the basis of high-resolution X-ray spectroscopic characterization, about half of the 147 phosphorus-rich regions examined in our samples were found to be polyphosphate, whereas the other half were classified as apatite, a common calcium phosphate mineral. From Diaz et al. [80]. Reprinted with permission from American Association for the Advancement of Science (AAAS) (Color figure online)

PolyP granules were detected in marine sediment, where they were mixed with granules of similar size that have been identified as apatite by X-ray fluorescence (Fig. 2) [80]. These apatite granules were theorized to originate from diatoms, as Diaz et al. [80] observed that the 0.5–3 μM polyP granules were similar in size to diatom granules. PolyP stores within diatoms are normally protected from the environment within their silica skeleton (frustule). Diaz et al. [80] proposed that after diatom death, bacteria consume the outer organic layer that protects the silica within the frustule, resulting in silica dissolution. After falling through the water column, polyP granules freed from or exposed within compromised frustules would be exposed to the sediment environment.

Although the exact chemical mechanism of granular diatom polyP depolymerization in the sediment is not understood, it was assumed that this polyP is the source of increased local Pi concentration, inducing apatite precipitation in the calcium-rich seawater that infuses the sediment [80]. It was suggested that polyP “appears to nucleate authigenic apatite growth” and “without extensive interaction with the free sedimentary phosphate pool.” This suggests that the P within the granule transformed into Pi that nucleated and formed an apatite mineral grain. The possibility that granules containing calcium and polyP could be transformed into apatite minerals echoes a theory from 1846 for bone mineralization [4]. This theory proposed that cells produce mineral precursor granules that are secreted into the ECM, where they transform into apatite granules.

## Mitochondrial Ca-/P-Rich Granules

In 1968, mitochondria were proposed to have a role in apatite biomineralization: concentrating intracellular calcium and Pi [115]. Ca- and P-containing granules were identified within the mitochondria of osteoclasts [116], chondrocytes [117, 118], osteoblasts [119], osteocytes [120–122], calcifying cartilage [123], and mineralizing bone [124–126] if samples were prepared with anhydrous or cryo techniques. Initial calcification loci, described as “roundish bodies of cellular origin” identified by Bonucci [127], led him to suggest that ions may have accumulated within mitochondria. Bonucci et al. [128] examined the ultrastructure of experimentally calcified mitochondria from various cell types, and found needle-shaped and roundish, electron-dense deposits, both showing an intimate relationship with an organic substrate having the same morphology as the inorganic deposits. Examination of rat liver mitochondrial granules after tissue microincineration resulted in the suggestion that they could be apatite precursors [129]. Incineration of bone-cell mitochondria produced unidentified “mineral” [122], while extracted mitochondrial granules after heating at 600 °C produced hydroxyapatite, Whitlockite, or a mixture of both [130].

In 1970, Lehninger wrote “We have adopted the working hypothesis. . . that what living cells “do” to Ca^2+^ and phosphate is to bring about their accumulation in the mitochondria to such concentrations as to exceed the solubility product of tricalcium phosphate, a process that cannot occur spontaneously in extracellular fluid, nor for that matter in the extramitochondrial cytoplasm. The end products of this stage are suggested to be “micro-packets” of insoluble amorphous tricalcium phosphate in the mitochondrial matrix, which we regard as the essential precursors of extracellular hydroxyapatite” [25]. This amorphous tricalcium phosphate theory has not been demonstrated. However, in 2012, electron-dense, noncrystalline, Ca- and P-rich granules were identified within, and possibly being transferred from, osteoblastic cell culture mitochondria processed with high-pressure freezing (HPF) and freeze substitution (FS) [131]. Similar nano-scale granules, with Ca/P ratios less than the 1.5 Ca/P ratio of amorphous calcium phosphate (ACP) [132], were detected in different conditions. The Ca/P ratio of ultracryomicrotomed mitochondrial granules in two chick tibias measured with high spatial resolution, nondispersive electron probe X-ray microanalysis were 1.04 ± 0.07 and 1.43 ± 0.14 [125]. Ca/P ratios of granules within undecalcified calcifying cartilage prepared under anhydrous conditions measured 0.8–1.1 [123], and the same ratio measured in mineralizing murine bone granules in cryo conditions was 0.75 ± 0.22 [133]. The Ca/P ratios of these granules are lower than those of tricalcium phosphate, hydroxyapatite, or ACP; but they are comparable with the Ca/P ratio <1 measured for Ca–polyP complexes by electron probe X-ray microanalysis [125].

## Ca- and P-Rich Granules in Apatite Biomineralizing Organisms

Intra- and extracellular apatitic granules, which have also been described by alternate names such as “spherules,” “spherulites,” “microspheres,” and “calcospherites,” were observed in calcifying cartilage [44–48, 118, 134–137], baleen [138], invertebrates such as the atremate brachiopods [45, 139], *Lingula adamsi* and *Glottidia pyramidata* [46], and the protozoa *Spirostomum ambiguum* [47, 140]. Watabe and Pan [46] commented that the atremate brachiopods could take up Pi from their diet and/or seawater and noted the “extremely high efficiency for phosphate accumulation” since the seawater contained much lower Pi concentrations. Electron microscopic images of brachiopods showed “the ability to maintain large storage of shell mineral components” as unstable “P- and Ca- containing granules” within the cells located in regions of primary and mineralized layers, as well as Ca- and P-containing precipitates in connective tissues [46]. Watabe and Pan [46] commented that the mechanisms of Ca and P accumulation, transport, and precipitation in the tissue were “virtually unknown.”

Jones [141] identified ^45^Ca and ^32^P uptake into endoplasmic “mineral deposits” in *Spirostomum ambiguum* and wondered if they might be similar to the 0.1–0.2 μm electron-dense granules previously observed in protozoan endoplasm [142]. Jones [141] suggested possible roles of these Ca- and P-containing granules, including P storage and an unexplained relationship with mitochondria. He noted that the granules were similar to dense granules observed in the mitochondria of osteocytes and osteoclasts.

**Fig. 3:**
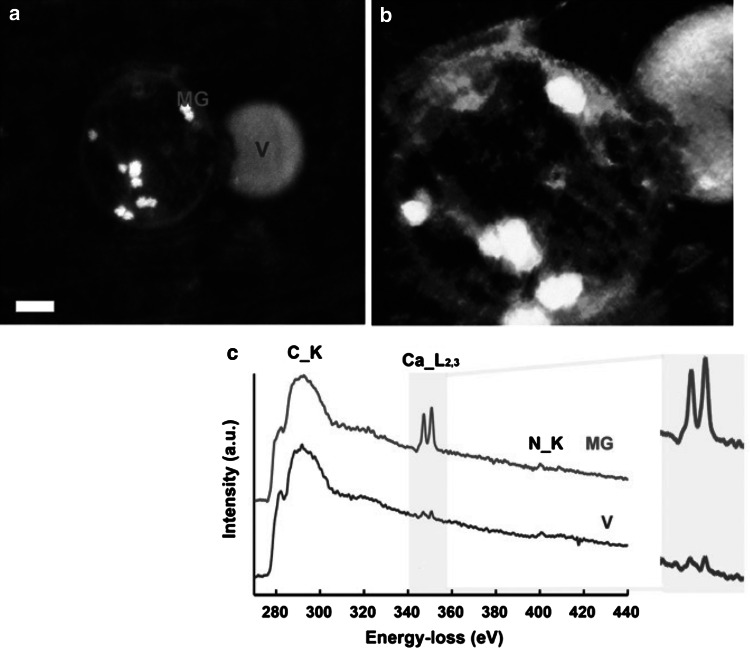
Analytical electron microscopic evidence of vesicle–mitochondrial interactions in mineralizing osteoblasts. **a** High-angle annular dark-field scanning TEM image of a dense granule-containing mitochondrion associating with a vesicle within an osteoblast in a mineralized nodule. The sample was prepared by high-pressure freezing and freeze substitution (HPF-FS). (*Scale bar* = 200 nm). **b** Voltex projection of a 3D tomographic reconstruction showing a mitochondrion conjoined with a vesicle. Dense granules are evident within the mitochondrion. Sample was prepared by HPF-FS. **c** Electron energy loss spectroscopy (EELS) of specified areas within the mitochondrion and vesicle in **a**. The mitochondrial granule and vesicle show characteristic calcium L2 and L3 edges at 346 eV. All spectra display carbon edges. **d** Orthoslices at 10-nm intervals through the tomographic reconstruction showing the mitochondrion–vesicle interface. The mitochondrial membrane is discontinuous where it conjoins the vesicle (*arrows*) [131]

Kashiwa and Komorous [135] demonstrated intra- and extracellular calcium- and P-rich spherules within fresh calcifying cartilage samples from regions preceding endochondral calcification. Kashiwa [143] also identified calcium- and P-rich granules within, and adjacent to, mature and hypertrophic calcifying chondrocytes when staining was performed on fresh samples to avoid the effects of sample preparation on unstable structures. Boonrungsiman et al. [131] observed Ca- and P-rich mitochondrial granules within mineralizing murine osteoblast cultures, and presented evidence of vesicle–mitochondrial interactions with high angle-annular dark-field scanning TEM of samples prepared with high-pressure freezing and freeze substitution (HPF-FS) (Fig. 3). Although the presence of these unstable, electron-dense, Ca- and P-containing granules has been identified by different groups, their specific composition is unknown. Fluorescence imaging has shown colocalization of polyPs within murine growth plate calcifying cartilage [31] but not at the resolution required to identify granules. How these granules are secreted into the ECM where they transform into carbonated apatite remains unknown. The realization of these phenomena must lie with the activity of matrix proteins.

## Revisiting the Secretory Theory for Biological Apatite Nucleation

In 1849, Quekett [4] postulated that organisms produce individual mineral precursor “granules,” which eventually became the building blocks of bone: “the granules being, in fact, the true ossific matter of the bone.” Watt [144] proposed a “secretory theory of mineralization” in 1928, arising from his observations of bone cells secreting granules that appeared to be incorporated into the mineralizing matrix. The theory that apatite mineral precursors were produced within mineralizing cells, processed through the Golgi, and exocytosed to the extracellular space where they are arranged into larger structures was reviewed in 1981 [145]. Weiner and Addadi [146] reviewed the vesicular strategy for four different biomineralization systems, including biological apatite.

## Biologically Controlled Apatite Nucleation In Vivo

Biologically controlled mineralization differs from biologically induced mineralization as it describes mineralization as a consequence of purposeful cell action. This action results in specific mineral sizes and chemistry within the extracellular matrices at specific locations and times [57]. Biologically controlled mineralization may proceed through the production of an intracellular biomineral precursor that is secreted into the ECM. Within the ECM, possibly within a granular microenvironment, matrix-mediated events initiate and control the transformation of the precursor into a mineral. Under biological control, that mineral may have a consistent size, composition, and mineralogy, irrespective of the mineral saturation state in the surrounding ECM. Simultaneous study of both the biochemical and physical chemical processes that initiate apatite biomineralization defies study by many analytical methods.

Bonucci [127] identified initial calcification loci within cartilage that were not associated with collagen, and reviewed the literature on the locus of initial calcification in cartilage and bone. He proposed that collagen fibrils are not the loci of initial calcification in cartilage, that ions may accumulate within mitochondria, and the earliest mineral precursors were found in “roundish bodies of cellular origin” for bone and cartilage. “Osteoblast extrusions” were found in the matrix between collagen fibrils, but their role in bone mineralization was unclear. Kashiwa and Komorous [135] also demonstrated intra- and extracellular calcium and phosphate-rich spherules within fresh calcifying cartilages preceding endochondral calcification. Bonucci reported the direct effect of sample preparation on newly mineralizing tissues, observing that bone crystals are individually surrounded by and integral with an organic phase (“crystal ghosts” [147]). This was observed only if the sample or section was decalcified after embedding [148].

The advantages and limitations of techniques used to examine biomineralization were reviewed by Bonucci [149]. He commented that many of the shortcomings of these techniques stem from the intimate inorganic and organic associations in bone that mask each other unless the sample is decalcified. Unfortunately, many decalcification processes disrupt the matrix [149]. This observation highlights one of the major challenges in studying biologically controlled apatite nucleation. In vivo biological apatite mineralization is controlled by proteins, while at the same time, the crystal nucleation must follow physical chemistry theory. The colocalization of matrix proteins with mineral nucleation may not always infer causation; however, enzyme activity must regulate biologically controlled apatite nucleation. Intracellular packaging of concentrated Ca and P stores with regulatory proteins that control apatite mineral nucleation and growth could describe the first step of a controlled biomineralization process. Ennever and Creamer [90] quoted Pautard, who made “an excellent point”: “It is unfortunate that the extensive investigation of collagen over the past few years has tended to obstruct serious survey of the nature of other organic substances associated with calcium salts in biological tissues. An almost universal preoccupation with collagen structure and chemistry has obscured the fact that there are . . . bone salts associated with other proteins and with polysaccharides” [150]. Glimcher ([151] citing [152]) summarized two theories of calcification: a “booster” theory, whereby an enzyme or group of enzymes cleaves Pi from an organic substrate, boosting the local Pi concentration, and the theory that the organic matrix induces apatite crystallization. The geology community has identified polyP as the “booster” process substrate in phosphorite formation. Could this Pi-boosting strategy also apply to biologically controlled apatite mineralization, which is ultimately controlled by the action of organic matrix components?

**Fig. 4:**
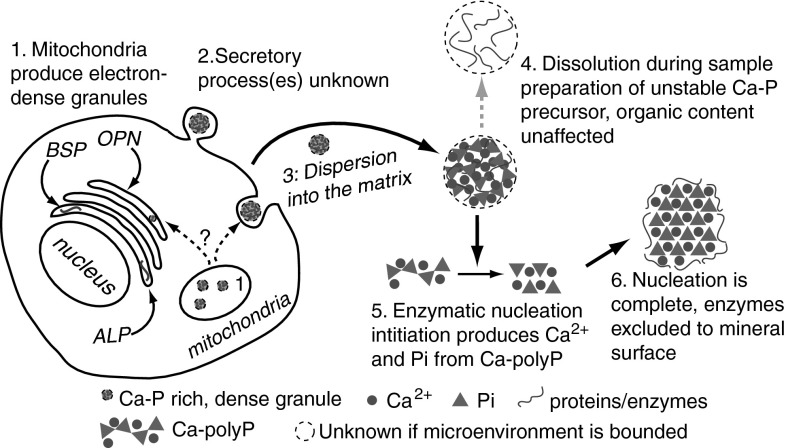
Proposed controlled apatite biomineralization schematic. (*1*) Mitochondria produce polyPs from phosphate sources that form complexes with calcium, producing discrete, electron-dense, Ca- and P-rich granules. (*2*) Granules may be processed through the trans-Golgi network (TGN), and then secreted via budding or exocytosis. If processed through the TGN, granules may associate with matrix and/or noncollagenous proteins as well as phosphatase enzymes. The secreted product is an amorphous Ca-/P-rich granule that contains matrix proteins. It is unknown if the granule is encapsulated. (*3*) The granule migrates to mineral nucleation sites within the collageneous matrix, where the noncollagenous proteins may play significant roles in granular interaction with the matrix. (*4*) During sample preparation, these unstable, amorphous granules may be artifactually dissolved so that only the remaining protein component is observed. These may be “crystal ghosts.” (*5*) If a phosphatase enzyme component of the unstable, amorphous precursor is activated within the matrix, the Ca–polyP component begins to transform into Ca^2+^ and Pi components. The local, high concentrations of Ca^2+^ and Pi nucleate apatite. As the apatite nucleus grows while the polyP depolymerizes, the protein component of the granule is excluded from the growing apatite crystal. This displaces the granule protein components to the surface. (*6*) The excluded proteins surround the apatite crystal surface, where they control crystal growth and shape, among other functions

Assuming that matrix proteins are closely associated with Ca–polyP within precursor granules, it is proposed that selective removal of Ca–polyP within the precursor granule would leave behind the organic components, which may describe the “crystal ghosts” (Fig. 4). Enzymatic initiation of apatite nucleation, by polyP depolymerization and increased Pi and calcium concentrations, would nucleate an ordered Ca–Pi structure within the amorphous granule. This nucleation of an ordered mineral would be expected to exclude the granular proteins from the nucleating apatite crystal lattice. This is because crystallization processes offer the phenomenon of purifying materials from an impure starting material [153]. As the apatite nucleus grows with more available calcium and Pi, it could exclude the associated granular proteins, eventually displacing them to the apatite mineral surface. When the granular polyP is consumed, the final product could be an apatite crystal now coated by the proteins that were secreted within the granule. This mechanism could provide one explanation for the transport of some proteins to skeletal mineral surfaces.

Minimal matrix disruption is a goal of cryo-electron microscopy (cryo-EM) and cryo sample preparation methods. Cryo-EM and HPF-FS preparation have recently provided evidence of a secretory process for bone mineralization [131, 133, 154, 155]. Mahamid et al. [133] imaged intracellular, membrane-bound, 80-nm-diameter Ca- and P-rich globules, which in turn are composed of smaller 10-nm spheres in mineralizing embryonic and up to postnatal day 2 murine bones. The nano-spherical subunits were “reminiscent of the intracellular and extracellular ACP nano-spheres dominating the newly-formed bones of the zebrafish fin” (citing [154, 155]). These nano-spheres were identified within preosteoblasts, osteoblasts, and osteocytes [133]. Electron-dense, extracellular, nano-scale granules within the cryosectioned, mineralizing ECM were not encased by membranes, and exhibited a laminated structure [156]. Mahamid et al. [133] suggested that osteoblasts secrete precursor mineral into extracellular mineralization sites, referring to previous evidence of intracellular mineral exocytosis in osteoblast cell culture [157]. Boonrungsiman et al. [131] also identified Ca- and P-rich granules within mineralizing cell cultures, and proposed a model for bone mineral formation “involving mitochondrial granules, calcium- and phosphorus-containing vesicles, and extracellular mineral precipitation.”

If it is assumed that polyP-containing granules are the source of calcium and Pi for apatite nucleation, the granular content could also control the new mineral volume. The size of diatom polyP granules was similar to the size of marine sediment apatite granules, and the polyP was theorized to represent the Pi content in the apatite granules [80]. Nano-sized bone mineral crystals were observed when isolated by the method of Weiner and Price [158]. Could an analogy be drawn for the formation of these skeletal mineral crystals from the Ca and P contents of secreted, nano-sized precursor granules?

## Conclusion

The orchestration of a controlled apatite biomineralization process represents an intricate and still unsolved mystery. This process requires the biological generation of apatite supersaturation levels to nucleate mineral for both biologically induced and controlled mineralization. Controlled apatite biomineralization processes also require cellular control of apatite nucleation initiation, possibly through the production of intracellular mineral precursors with concentrated calcium and phosphorus stores, and further biological control of matrix-mediated mineral nucleation events.

Challenges in apatite biomineralization continue to be addressed by scientists in the geological, biological, pathological, and medical sciences. Recent advances in geomicrobiology have shown that the biological concentration of Pi from an aqueous environment can be polymerized by mitochondria and stored as polyP. It has been demonstrated that polyP can serve as a concentrated Pi source, resulting in the controlled, extracellular release of Pi by living bacteria. This release into the local environment increases calcium phosphate mineral saturation, leading to biologically induced phosphorite nucleation. It is possible that oral bacteria with polyP stores may induce dental calculus formation with a similar chemical mechanism.

Within marine sediment, the depolymerization of Ca–polyP granules from diatoms may transform into discrete apatite granules. The identification of calcium- and P-rich granules in the biologically controlled, apatite biomineralizing protozoa, inarticulate brachiopods, and the mineralizing vertebrate skeleton suggest a Ca- and P-concentrating mechanism involving polyP. PolyP has not been identified in these granules, but the low Ca:P ratio in vertebrate skeletal amorphous granules suggests its presence. The controlled apatite biomineralization literature contains evidence of the production and secretion of electron-dense, unstable, Ca- and P-containing granules; but their association with apatite nucleation events that are controlled by matrix proteins remains speculative.

It is a paradigm shift in geology that phosphorite-producing organisms use a polyP intermediate to concentrate Pi. Could this paradigm shift apply to controlled apatite biomineralization? It may be useful to integrate the geological perspective of polyP-induced mineralization, the physical–chemical theories of crystal nucleation, and the critical role of organic matrix proteins to understand the complex biological events in calcified tissue formation.
